# FAP Associated Papillary Thyroid Carcinoma: A Peculiar Subtype of Familial Nonmedullary Thyroid Cancer

**DOI:** 10.1155/2015/309348

**Published:** 2015-12-01

**Authors:** Francesco Cetta

**Affiliations:** IRCCS MultiMedica, Viale Fantoli 16/15, 20138 Milan, Italy

## Abstract

Familial Nonmedullary Thyroid Carcinoma (FNMTC) makes up to 5–10% of all thyroid cancers, also including those FNMTC occurring as a minor component of familial cancer syndromes, such as Familial Adenomatous Polyposis (FAP). We give evidence that this extracolonic manifestation of FAP is determined by the same germline mutation of the APC gene responsible for colonic polyps and cancer but also shows some unusual features (F : M ratio = 80 : 1, absence of LOH for APC in the thyroid tumoral tissue, and indolent biological behaviour, despite frequent multicentricity and lymph nodal involvement), suggesting that the APC gene confers only a generic susceptibility to thyroid cancer, but perhaps other factors, namely, modifier genes, sex-related factors, or environmental factors, are also required for its phenotypic expression. This great variability is against the possibility of classifying all FNMTC as a single entity, not only with a unique or prevalent causative genetic factor, but also with a unique or common biological behavior and a commonly dismal prognosis. A new paradigm is also suggested that could be useful (1) for a proper classification of FAP associated PTC within the larger group of FNMTC and (2) for making inferences to sporadic carcinogenesis, based on the lesson from FAP.

## 1. Introduction

Familial Nonmedullary Thyroid Carcinoma (FNMTC) is a nonmedullary thyroid cancer occurring in a subject with germline mutation for a gene responsible for an inherited multitumoral syndrome, including thyroid carcinoma as a part of the syndrome (or in more than 3 members of the same kindred, even in the absence of a known mutation in a putative gene). FNMTC makes up to 5–10% of all thyroid cancers, also including those FNMTC occurring as a minor component of familial cancer syndromes, such as Gardner's syndrome, Cowden's disease, Carney complex type-1, Werner's syndrome, McCune Albright syndrome, or Familial Adenomatous Polyposis (FAP) [1, 2]. In particular, a recent review outlines that “FNTMC is associated with more aggressive disease than sporadic cases, with higher rates of multicentric tumours, lymph node metastasis, extrathyroidal invasion, and shorter disease-free survival” [1].

In addition, it has been suggested that “the genetic inheritance of FNMTC, (in patients in whom it is the predominant feature), remains ‘unknown…'”, but “it has been observed an increased percentage of male patients with FNMTC compared to those with sporadic NMTC” [1].

We have collected the largest world series of patients with FAP (Familial Adenomatous Polyposis) associated FNMTC (*n* = 18, all females) and were the first to show a significant genotype-phenotype correlation, suggesting that most of APC (Adenomatous Polyposis Coli) germline mutations in patients with FAP associated FNMTC are located in a well defined genomic area, that is, 5′ to codon 1200 [3–17].

FAP is inherited in an autosomal dominant fashion and is characterized by multiple adenomatous polyps in the colon and rectum and a near certainty of developing colorectal cancer unless a risk-reducing prophylactic colectomy is performed. Therefore, early identification and intervention in FAP patients is of paramount importance. FAP is also associated with several extracolonic malignancies, including malignancies of the upper gastrointestinal tract, hepatobiliary tract, central nervous system, and endocrine system (thyroid, adrenal) [3–10].

While many individuals are diagnosed with FAP due to the discovery of colonic polyps, the identification of CMV (cribriform-morular variant) histology in patients with PTC provides clinicians with a unique opportunity to diagnose occult cases of FAP [18–25].

During the last decade we have continued (1) to recruit FAP associated FNMTC, (2) to follow up those previously collected, and (3) to collect all available data from the literature [11–17, 26].

## 2. Female Prevalence

In our review of the various series (often including just 1 or 2 cases) we could document that whereas in our first report on FAP associated FNMTC, the prevalence for females was 17 : 1, after 2000, there was a striking increase (female to male ratio = 80 : 1 versus 2.5–3.1) in sporadic tumors [11–17, 26].

## 3. Age at Diagnosis of PTC and/or Colonic Polyps

More than 80% of FAP associated FNPTC were diagnosed between 18 and 35 years of age. In particular, in our 18 patients, diagnosis was concomitant in 6 (1/3), whereas in 6 (1/3) FAP preceded and in 6 (1/3) PTC preceded. This is very important, because the early occurrence of PTC can facilitate diagnosis in some kindred with undetected FAP [5, 16].

## 4. Long-Term Prognosis

In an overall series of 200 cases reported in the literature (112 before 2000 and 90 after that year), there were very few recurrences and only 1 death, possibly related to FAP associated PTC [16, 17, 26]. In particular, there was no recurrence in 9 of our patients, with a follow-up longer than 15 years (180 months) in every subject [26].

## 5. Prevalence of PTC in FAP Patients

Concerning the actual prevalence of PTC in FAP patients, it has been reported as 0.4% to 2% in various retrospective series. More recently, results of prospective registry screening programs for PTC in patients with FAP have reported prevalences of 2.6% [27] to 11.8% [28]. This is an increased value in comparison with the previously reported data (1.2%) [23]. We deem that a 3 to 5% prevalence of PTC in FAP patients could be a more realistic value in the present era of improved early diagnosis [26]; even if recent intensive screening protocols in patients belong to FAP registries, the prevalence was 6.1% overall and 11.1% in women [29, 30].

The main criticism that can be raised in the interpretation of the findings of the last series, in which all FAP patients underwent intensive screening, concerns the significance of observed data. In fact, in some cases thyroid nodules resulted positive at FNAB, even in males and after age 60. This observation contrasts with previous observations, suggesting that these tumors are likely to be different from those currently associated with FAP, occurring in females aged less than 35.

## 6. Age of Patients

In our first report the mean age of patients was 24.8 years, in a series of 15 female patients [5], but it was also 24.8 years in a series of 97 patients collected from the literature. The mean age has been similar in the 81 patients reported in the literature after 2000 [26].

## 7. Histologic Variants of PTC

Harach et al. [19] reported 4 cases of thyroid carcinoma in FAP patients and noted the following unusual features: multifocality (differing from sporadic PTC) multifocality-tumors encapsulated, and unusual histologic patterns: cribriform, solid, spindling, and whorls. They concluded that FAP associated thyroid carcinomas were likely related to PTC but were sufficiently different.

Cameselle-Teijeiro and Chan [20] described 4 cases with similar or identical morphology, apparently occurring in patients without FAP. These findings were also observed by others [21]. These tumors have been termed the “cribriform morular” variant of PTC (CMV PTC). The cribriform areas are composed of anastomosing bars and arches of cells without intervening stroma with the follicular spaces devoid of colloid. The morulas are composed of spindled cells with “peculiar nuclear clearing.” These clear nuclei differ from both the optically clear nuclei and intranuclear pseudoinclusions more typically seen in PTC and consist of accumulation of biotin [20–22].

Harach et al. [19] speculated that the morphology of this variant could be related to the involvement of the APC gene in the pathogenesis. However, we never found balletic inactivation in the thyroid tumoral tissue (that was found in the colonic tumoral tissue); Soravia et al. [25] in 9 samples from 4 patients found somatic APC mutations in 1 sample. These findings suggest that although somatic mutations of APC may be seen in a few cases of FAP associated thyroid carcinoma, it is not a required step in the pathogenesis of these neoplasms. On the contrary, we were the first to show a very high incidence of ret/PTC oncogene activation, which is known to be an early molecular event in papillary thyroid carcinoma oncogenesis [7]. These findings support the concept that FAP associated thyroid tumors are variant of PTC [7]. In a subset of CMV of PTC Xu et al. [22] have demonstrated that aberrant nuclear accumulation of mutant beta-catenin may substitute for APC mutations. It is thought that these sporadic cases are due to a somatic mutation in exon 3 of the beta-catenin gene (CTNNB1), further highlighting the analogous role to the APC-beta-catenin pathway. In fact, not only sporadic CMV PTC but also FAP associated PTC usually, but not always, show nuclear and cytoplasmatic expression of beta-catenin [12, 16, 26].

On the contrary, the most common genetic abnormality in papillary thyroid carcinoma BRAF mutation appears to be absent in FAP associated thyroid cancer, suggesting that BRAF and APC RET/PTC mutations are mutually exclusive of each other in the occurrence of different types of PTC [16, 17, 26].

However, there are some clinical, demographic, and prognostic differences between sporadic CMV-PTCs, which may occur in males (instead of exclusively in females) and in old age, and many reports show an aggressive behavior with distant metastasis [20, 21, 29] and the typical indolent behavior of FAP associated PTC, occurring almost always in females and showing an indolent behavior [5, 26]. There was only 1 death because of thyroid related complications out of 200 FAP associated PTCs [26].

Therefore, we suggest caution before stating that, despite similar features and the possibility of diagnosing CMV PTC preoperatively by FNAB, these tumors (sporadic and FAP associated) should be considered as a single entity [16, 17].

Interestingly, whereas tumors in the colon rectum occur invariably in almost 100% of subjects with APC germline mutations, with no prevalence for any sex and with the same incidence in males and in females (and in most colorectal polyps or cancers, there is a complete loss of the APC function documented by the high rate of LOH for APC in the tumoral tissue), FAP associated FNMTC occurs in a minority of affected siblings, in the absence of LOH for APC [6] and almost invariably in the female sex [26]. This is opposite to what has been reported for FNMTC as a homogeneous entity [1], since these authors observed a relative prevalence of males in comparison to the usual F : M ratio = 3 : 1 in sporadic tumors.

In particular, this is a very unusual finding in an inherited multitumoral syndrome. There is no doubt that FAP associated FNMTC is part of the multitumoral syndrome due to germline mutations of a tumor suppressor gene as APC. In fact, there is a frequent association of FNMTC in siblings with the same germline mutation (all the 23 siblings with FAP associated FNMPC were female).

There is a statistically significant association between FNMTC and the site of germline mutations (almost all mutations are located in the proximal portion of the gene, 5′ to codon 1220) [5].

However, the absence of complete inactivation of the gene [6] suggests that the germline mutation of the APC gene confers only a generic susceptibility to thyroid cancer [5–11], but perhaps other factors, namely, modifier genes, sex-related factors (hormonal, but also dietary, metabolic, and immunological), or environmental factors [31], are also required for the phenotypic expression.

It is likely that FAP associated FNMTC represents a veritable example of cooperation between purely inherited factors (APC germline mutation) and epigenetic or environmental factors, namely, those strictly connected with the female sex, as the striking female to male ratio of 80 : 1 strongly suggests [26].

These peculiar features have been documented in detail only for FAP associated FNMTC, with the striking female preponderance.

In FNMTC associated with Werner syndrome, a relative male prevalence has been suggested by some authors [1]; as well in other FNMTC associated with other inherited multitumoral syndromes there could be other associations with epigenetic or environmental factors [1].

We suggest that a similar multifactorial cooperation could also occur for the most frequent variant of FNMTC that is not associated with a germline mutation of a known tumor suppressor gene. We suspect that the 5–10% of FNMTC do not include just a few rare manifestations of 3-4 inherited multitumoral syndromes (such as those quoted in a recent review) [1], whereas the remaining patients belong to a unique disease, for which (in addition to a common prognosis or similar biological behavior) also a single predisposing gene or a common molecular or pathophysiologic mechanism should be envisaged. This is an oversimplification of a complex entity.

Actually, Navas-Carrillo et al. [1] report (1) on the American family with 5 members affected by PTC, one by colon cancer and 2 by papillary renal neoplasm, with a possible susceptibility gene (PTC/PRN), located at 1q21 [32], (2) on the susceptibility locus (NMTC1) on chromosome 2q21, firstly identified in a large Tasmanian pedigree with recurrence of PTC [33], and (3) on the susceptibility locus to chromosome 8p23.1-p22 in a large Portuguese family with 11 cases of benign thyroid disease and 5 cases of thyroid cancer [34]. In particular, patients with these genetic alterations may partially overlap with, or be completely different from, those showing genetic anticipation (i.e., diagnosis at an earlier age in patients of the second generation) [35].

Anyway all these patients are smaller in number than the 200 homogeneous patients with FAP associated NMPTC, with documented association with an inherited germline mutation, for whom we have shown a biological behavior (striking female prevalence and little tumor aggressiveness) different from that suggested by Navas-Carrillo et al. [1] for the total amount of FNMTC.

On the basis of these cumulative data, it is more likely that there is a galaxy, a wide multiplicity, of potential germline mutations, each of which can confer an increased susceptibility, but other genes or factors, namely, environmental factors (maybe playing a greater role than congenital predisposition), are also required for PTC manifestation.

This great variability of germline predisposing factors and of epigenetic and environmental factors is against the possibility of classifying all FNMTC as a single entity, not only with a unique or prevalent causative genetic factor, but also with a unique or common biological behavior, such as “higher rates of metastases, extrathyroidal invasion, and shorter disease free survival.”

The only common characteristic (in addition to the possibility of earlier diagnosis, because of a previous affected sibling in the same kindred) is the frequent multicentricity. But, also in this respect, it must be outlined that we had no recurrence in the contralateral lobe (after a minimum follow-up of 180 months for all patients), in 5 out of 5 subjects with FAP associated FNMTC, who had hemithyroidectomy, because they refused total thyroidectomy, in association with total colectomy and other concomitant invasive operations at a young age, which are usually required in FAP subjects [16, 17, 26].

In particular, there is no single genetic alteration predisposing specifically to FNMTC in all cases. On the contrary, there are various syndromes, also including some familial cancer syndromes, that have multiple siblings with FNMTC as a part of the syndrome.

Therefore, if FNMTC is a very heterogeneous syndrome, to try to select common features as well as a uniform prognosis or the same biological behavior can be misleading.

Furthermore, the lesson from the galaxy of heterogeneous FNMTC, if correctly interpreted, could contribute to open new avenues for a deeper knowledge of pathogenic factors determining the occurrence of all “common” or most frequent types of cancers.

From a more general point of view, concerning inherited predisposition of tumors, we hypothesize that, concerning genetic predisposition, in the vast majority of “common cancers” (such as lung, breast, cancer, pancreas, and liver, also including thyroid cancer), there is an individual way to cancer occurrence; that is, there is a single gene or an oligogenic alteration, that is, one or more germline mutations occurring in some cancer facilitating or controlling genes, but also in genes controlling immune response or other functions, which predisposes to cancer. This cancer predisposition that is not due to a single germline mutation in a tumor suppressor gene (such as in the rare inherited multitumoral syndromes), but due to a combination of genes, could segregate more frequently in siblings, facilitating the familial occurrence of some cancers [36].

In particular, our recent studies on oligogenic germline mutations in nonsmoker-discordant siblings with lung adenocarcinoma (1) confirmed, as a “proof of concept,” the hypothesis of an oligogenic combination for cancer susceptibility, (2) further support a model of “private genetic epidemiology” for a better understanding of the genetic effects in families with common cancers, and (3) suggest the possibility that each individual may have his/her personal way to cancer. These findings could have important implications for personalized medicine [36]. It is noteworthy that, for thyroid cancer, the common exposure to radiation [31] or other environmental factors to which siblings living in the same site could be exposed, in association with inherited predisposition from a wide range of susceptibility genes, should also be taken into account [26].

## 8. Conclusion and Lesson from FAP Associated PTC to Familial and Sporadic Carcinogenesis

1. Concerning FNMTC, the preliminary knowledge of the familial aggregation of a given cancer could be a useful tool for early tumor detection. But this should not be used to conclude that FNMTC should be considered as a single entity, a unique disease, with a common pathogenic mechanism.
2. We must recognize that, on the basis of present data, we are unable to answer uniformly to the question as to whether FNMTC is more or less aggressive than its sporadic counterpart. The correct answer could be “in some cases yes, in others no.” It depends on inherited genetic alterations facilitating cancer susceptibility, but also on the individual tumor of a given subject.
3. We suggest that time has come that, on the basis of current clinical evidence, we must challenge and also try to confute the “old scientific paradigm” and to provide a “new paradigm” that is more in accordance with actual genetic, pathologic, and clinical evidence. After the “genetic revolution” following DNA discovery and human genome sequencing, together with the observation that a single base change in a single gene (as a germline mutation) could be responsible for the occurrence of a given cancer in 100% of affected subjects (such as in FAP), the utopian dream has been cultivated that “targeted genetic engineering” could cure clinically evident cancer. Unfortunately, this is not the case. Analogously, clinical evidence and long-term follow-up (as in FAP associated FNMTC or in MEN 2A associated endocrine tumors) have shown that it is not true that a tumor belonging to an inherited multitumoral syndrome (even if frequently multifocal) has a more aggressive behavior and a worse prognosis than its sporadic counterpart.
4. Not only many multitumoral syndromes (such as FAP), but also nontumoral diseases (Alport syndrome, etc.), instead of being determined by a single germline mutation in a single gene, may be determined not only by mutually exclusive mutations in different genes (APC and MYH) [37], but also by concomitant mutations in multiple genes, so determining digenic diseases (with at least 2 pathogenic germline mutations in at least 2 of the 3 genes COL4A3, COL4A4, and COL4A5, responsible for Alport syndrome) [38] or even by somatic mosaicism [39]. In summary, things and tumors have a greater complexity than what was initially hypothesized. Better classification and grouping of similar diseases can be useful, but incorrect grouping (mixing apples and oranges) can be misleading [36].
5. The new paradigm can be the following. A single genetic mutation, but also, and perhaps more frequently, an oligogenic group of germline mutations (in some cancer related genes, but also in other genes) sometimes overlapping among individuals, but often differing from one individual to another, can be responsible for cancer (or common diseases) predisposition, together with a wide range of epigenetic or environmental factors (the “weight” of which can also be greater than that of congenital predisposition), or necessary for its full phenotypic manifestation. In addition, biological behavior, aggressiveness of the tumor, and also susceptibility to tumor occurrence, do not depend only on the quantity or the “potential danger” of the “offending agent,” but also on the resistance of the subject, not only at the level of the targeted cell or tissue, but also as “host resistance” as an entire “indivisible” organism [36].
6. The true challenge of the near future is that available data from the literature can be used and interpreted according to the “old paradigm,” considering FNMTC as a single entity, with a common biological behavior, or according to the “new paradigm” as “a galaxy of different diseases,” each one with its peculiar combination between congenital predisposing factors, also facilitating familial aggregation, and environmental factors, determining in every patient a “unique type of cancer.” This new paradigm fits more and is more in accordance with the so-called “personalized medicine” [36].
